# Beyond the Individual: Social and Cultural Influences on the Health-Seeking Behaviors of African American Men

**DOI:** 10.1177/1557988319829953

**Published:** 2019-02-15

**Authors:** Natalie T. Eley, Emily Namey, Kevin McKenna, Annette Carrington Johnson, Greg Guest

**Affiliations:** 1 FHI 360, Durham, NC, USA; 2 North Carolina Central University, Durham, NC, USA

**Keywords:** African American men, health-seeking behaviors, social influences, culture, focus groups

## Abstract

Morbidity and mortality rates are alarmingly high among African American men and are influenced by the health-seeking behaviors of this population. This study examined data from 40 focus groups with African American men in Durham, North Carolina, to better understand social and cultural influences on health-seeking behaviors. Data were analyzed using inductive thematic analysis. Three broad types of social/cultural influence on motivation to seek health care services were identified: family, culture and upbringing, and peers. Study findings confirm the importance of social relationships in influencing African American men’s health-seeking behaviors and offer characterization of the nature of influence across different types of relationships, according to the direct support or indirect messages they provide. Future programs can draw on these data to inform efforts to include family and peers as well as utilize existing cultural gender norms to the advantage of health promotion for African American men.

African Americans represent an underserved population in terms of many key health indicators in the United States (Centers for Disease Control and Prevention, 2013) and in North Carolina (Centers for Disease Control and Prevention, 2013; Office of Minority Health and Health Disparities & State Center for Health Statistics, 2010). African American men, in particular, are less likely than other groups to seek treatment or preventive health care services (Spalter-Roth, Lowenthal, & Rubio, 2005; Welch, 2003). African American men are unwilling to seek medical attention due to prevailing norms in the African American community (Griffith, Allen, & Gunter, 2011). As Williams observes about men in general,

Beliefs about masculinity and manhood that are deeply rooted in culture . . . play a role in shaping the behavioral patterns of men in ways that have consequences for health. Men are socialized to project strength, individuality, autonomy, dominance, stoicism and physical aggression, and to avoid demonstrations of emotion or vulnerability that could be construed as weakness. (Williams, 2003, p. 726)

These norms can translate into fewer encounters with the health care system, delayed attention to symptoms, poor medication compliance, and an unwillingness to talk openly about health concerns (Liburd, Namageyo-Funa, & Jack, 2007). For example, Liburd et al. (2007) conducted a thematic elaboration of data from illness narratives of a sample of African American men living with Type 2 diabetes, with a focus on masculinity and its connection with challenges of diabetes self-management. Their findings include men wanting to maintain control over their care and bodies despite medical consequences and placing limits on the social support they accept. In another study, Hawkins and colleagues (2017) conducted focus groups with African American and Latino men with Type 2 diabetes to explore gender values and beliefs within these specific groups and how these influenced their health behaviors. Their findings highlight participants’ beliefs about manhood and illustrate how the desire to maintain control over their own health (e.g., keeping a “strong image” among others and being hesitant to follow health advice) hinders their health-seeking behaviors (Hawkins et al., 2017). Both of these studies suggest that masculine gender identity is a cultural factor that influences the health-related behaviors of African American men (Hawkins et al., 2017; Liburd et al., 2007).

Other research specifically focused on African American men has identified barriers to and facilitators of their decisions to seek health care, particularly with regard to preventive screening procedures (Plowden & Miller, 2000; Plowden & Young, 2003) such as prostate examination (Blocker et al., 2006; Jones, Steeves, & Williams, 2010; Myers et al., 1996; Plowden, 2006; Woods, Montgomery, Belliard, Ramirez-Johnson, & Wilson, 2004). There are, of course, structural and historical factors that affect the health-seeking behaviors of African American men as well, such as accessibility of resources (Plowden & Miller, 2000; Plowden & Young, 2003), racism (Boyd, 2018; Gee, 2016; Gee & Ford, 2011; Hall, Hall, & Perry, 2016; Hartfield, Griffith, & Bruce, 2018; Jee-Lyn García & Sharif, 2015; Williams& Williams-Morris, 2000), mental health/stress (Holden & Xanthos, 2009; Williams & Williams-Morris, 2000), medical mistrust (Blocker et al., 2006; Cheatham, Barksdale, & Rodgers, 2008; Hammond, Matthews, Mohottige, Agyemang, & Corbie-Smith, 2010), and socioeconomic status (Cheatham et al., 2008), to name a few, including the intersectionality of factors that contribute to health inequalities for African American men (Griffith, 2012; Griffith, Ellis, & Allen, 2013; Lewis & Van Dyke, 2018). Separately from society-level structural factors, many studies report interpersonal-level influences on health-seeking behavior. These include spouses/partners, family, and friends (Blocker et al., 2006; Jones et al., 2010; Myers et al., 1996; Plowden, 2006; Plowden & Young, 2003; Ravenell, Johnson, & Whitaker, 2006; Woods et al., 2004), faith and religious leaders (Avent & Cashwell, 2015; Avent, Cashwell, & Brown-Jeffy, 2015; Blocker et al., 2006), providers (Jones et al., 2010; Myers et al., 1996), and the type of male socialization referenced by Williams (Blocker et al., 2006; Plowden & Young, 2003).

Despite this evidence that socially related factors have an impact on African American men’s health-seeking behavior (Cheatham et al., 2008), few studies drill down into the particulars of social influence, beyond that it is provided by family, friends, and significant others. Greater clarity on the mechanisms and effects of these types of social relationships in relation to African American men’s health will allow the field to capitalize on social influences that promote positive health-seeking behaviors (Berkman, 1995). This analysis adopts the definition forwarded by Glanz, Rimer, and Viswanath (2008) defining social support as conscious aid and assistance exchanged through social relationships and interpersonal transactions and inclusive of the concept of social influence as the process by which thoughts and actions are changed by observing the actions of others. Use of the term “social influences” reflects the effects of interpersonal communication and behavior observation. Using this definition of social influence as a guide, this article draws on qualitative data from a study conducted among African American men in North Carolina to describe in detail the types and means of social influence exerted by family, peers, and culture as a way to identify specific points for sociocultural interventions for health promotion.

## Methods

### Data Collection

Data for this analysis are drawn from 40 focus groups conducted with African American men in Durham, North Carolina, between January and May 2013 as part of the Durham Focus Group Project (DFGP). The DFGP was a qualitative methodological study on the topic of African American men’s health-seeking behaviors (methods findings described elsewhere; Guest, Namey, & McKenna, 2017). Because of the participant dynamics they engender, focus groups are often used to learn about the social and normative knowledge that underpins participants’ life experiences (Guest, Namey, & Mitchell, 2013; Krueger & Casey, 2015), making them a relevant approach for this research. Participants were recruited through a combination of local stakeholder networks (including religious and community organizations), Craigslist, and flyers posted in public areas and health clinics in the downtown Durham area. A draft of the focus group instrument was pretested among a group of five men from the target population and revised based on their responses. The final instrument contained 13 open-ended questions that pertained to men’s experiences with and perceptions of health behavior and health care (Guest et al., 2017). For uniformity in data collection, one experienced data collector facilitated all the focus groups with an assistant present to administer informed consent and aid with logistics. Before initiating the focus groups, verbal informed consent was obtained from all participants individually after an oral consent form was read to them. The data collector signed and filed a de-identified consent form for each focus group participant, noting the date and time of participation. The data collector verbally confirmed informed consent at the beginning of each focus group recording. Each focus group lasted approximately 2 hours and study participants were provided an incentive of US$40 for their time. The study was reviewed and approved by FHI 360’s Protection of Human Subjects Committee (Study Number: 10363).

### Data Analysis

Focus groups were digitally recorded and professionally transcribed verbatim, following a detailed transcription protocol (McLellan, MacQueen, & Niedig, 2003). After verifying the accuracy of the transcripts, they were imported into NVivo Version 10 (QSR, 2012) to facilitate coding and analysis. A systematic inductive thematic analysis (Guest, MacQueen, & Namey, 2012) approach was used, in which themes and codes are generated from the data. The coding process is exploratory and occurs without trying to fit the data into a preexisting model or frame. Two analysts (including the data collector) read each transcript independently and then agreed on themes to be formalized as codes. Once themes were formalized, a thematic codebook defining all codes was created following a team-based iterative process (MacQueen, McLellan-Lemal, Bartholow, & Milstein, 2008). Analysts used NVivo Version 10 (QSR, 2012) to independently apply codes to segments of text in each transcript. After coding each transcript, the two analysts met to compare code application and discuss new emergent codes. All discrepancies were resolved through discussion, resulting in a consensus coded document.

At the completion of all coding, written summaries were generated for each code related to social or cultural influences on health behavior to identify patterns within and across coded material. Through collective discussion and analysis of these written summaries, final themes were identified and categorized by specific type of social or cultural influence to address the research objectives.

## Results

This study recruited a total of 310 African American men from Durham, North Carolina, to participate in the 40 study focus groups. The focus groups included 6 to 8 individuals and averaged 7.75 individuals each. The sample ranged substantially in age, with a mean of 47 years (Table 1). The majority of men had completed high school (205; 67%), were unemployed (234; 78%), and had an annual household income of less than $20,000 (235; 78%). Although most of the men had seen a physician within the past year (217; 70%), many of them did not currently have health insurance (151; 61%).

**Table 1.** Focus Group Participant Demographics.

Demographics^a^	
Age (*n* = 309)	
Mean	47
Range	25–65
Highest level of education (*n* = 306)	
Elementary school	3 (1.0%)
Middle school	15 (4.9%)
High school	205 (67.0%)
Associate degree/tech college	46 (15.0%)
BA/BS	27 (8.8%)
Graduate degree	5 (1.6%)
Other/trade school	5 (1.6%)
Employment status (*n* = 299)	
Employed	65 (21.7%)
Unemployed	234 (78.3%)
Retired	1 (0.3%)
Annual household income (*n* = 299)	
<$20,000	235 (78.6%)
$20,000–$40,000	43 (14.4%)
$40,001–$60,000	15 (5.0%)
$60,001–$80,000	4 (1.3%)
>$80,000	2 (0.7%)
Health insurance status (*n* = 247)	
Private	22 (8.9%)
Medicaid	48 (19.4%)
Veterans benefits	19 (7.7%)
More than one type of insurance	7 (2.8%)
Uninsured	151 (61.1%)
Has primary care physician (*n* = 242)	
Yes	125 (51.7%)
No	117 (48.3%)
Has seen a physician in past 12 months(*n* = 307)	
Yes	217 (70.7%)
No	90 (29.3%)

a The *n* varies for some questions due to nonresponse. Overall *N* = 310.

This analysis focused on the thematic codes related to the overarching construct of social and cultural influences on health behavior. Ten such thematic codes were identified and defined (see Table 2). Subsequently these thematic codes were organized into three broad domains of influence, as aligned with the literature, Family, Peers, and Cultural and Gender Norms, and further analyzed thematically according to the specific motivating (or demotivating) influence on African American men’s health-seeking behaviors. In this analysis, family is defined as spouses, children, parents, grandparents, siblings, and other extended relatives. The domain “Peers” comprises friends, associates, and other members of the community.

**Table 2.** Thematic Codes and Definitions.

Code name	No. of FGs	Abridged definition: Use this code for discussion of …
***Peer experiences***	40	. . . peer experiences—what happened to one’s friends or family members—as a factor affecting whether and how one seeks health care.
***Pride/male gender roles***	40	. . . men being too proud to see a provider, or thinking they are less of a man/weak if they seek care for any ailments, as a factor affecting whether or how one seeks health care. Includes discussion of male gender roles around health and health care and terms like machismo, ego.
***Upbringing/how we came up***	40	. . . upbringing or how one was raised as a factor affecting whether and how one seeks health care.
***If it ain’t broke/I’m all right***	38	. . . belief that if there’s not something seriously broken, there’s no need for medical attention, often summarized as “if it ain’t broke, don’t fix it,” typically as a factor affecting whether and how one seeks health care.
***Home remedies***	37	. . . home remedies, alternative, or naturopathic medicine accessed or applied outside of the standard biomedical system.
***Family history***	35	. . . family history or susceptibility to a disease or condition; genetic predisposition or heredity as a factor that affects whether or how one seeks health care.
***Family motivates***	30	. . . family members urging men to seek health care as a factor affecting whether and how one seeks health care. Also use for men wanting to take care of their health for the benefit of their family.
***Invincibility***	29	. . . invincibility or a perception of immunity to risk or hazard as a factor affecting whether or how one seeks health care.
***Celebrities/famous role models***	15	. . . celebrities (local or national) or other famous or well-known role models as they relate to health, health beliefs, or health care.
***Social pressure/influence***	9	. . . social pressure or influence—positive or negative—that affects whether and how one seeks health care.

*Note*. FG = focus group.

Thematic codes related to the social and cultural influences on health- and health care–seeking behavior of African American study focus group participants.

### Family Influences on Motivation to Seek Health care Services

Female partners—referred to as a wife, lady, girlfriend, and significant other—were most frequently cited as a source of family influence on men’s health-seeking behaviors, followed by children and parents. Though a few of the men in the sample discussed having a male partner, none spoke of their male partner as a strong influence on their health-seeking behavior; therefore, in this analysis, the word “partners” refers to female partners.

#### Partners

Female partner influence on health-seeking behaviors was mentioned in 19 of 40 (47%) focus groups. Men often noted that if female partners did not encourage them or “force them” to get medical attention, then men would not seek medical care for themselves. Partners’ encouragement of men took the forms of verbal expression and/or simple presence, in that men perceived an obligation to be alive and healthy for their partners. Female partners’ motivation and encouragement of men’s health-seeking behaviors were discussed in a positive light; the men seemed to appreciate both the concern their partners showed and the “out” they were given by having someone else suggest they seek medical attention.

So usually it’s the wife, the girlfriends, “When’s the last time you had a check-up? Don’t you have a check-up next week? You need to go.” So a lot of time it takes that outside influence to encourage us to do it because . . . we men. We not just black men, we men. So we tough. There’s a role we have to play. There’s a façade that we have to wear to let our women, let our children know we’re men. (FG07; age 54)

The only thing that got me in there [surgery] is my wife was standin’ right there and I said, “Well baby, you the first thing I’m lookin’ at when I go under here . . . [that] I wanna see when I come [out]. I don’t want you to go nowhere.” But she had to leave of course through the surgery. But that’s just me. I think on the outside I’m this big tough guy, but when it’s time for the doctor, you can forget that. I ain’t going to no doctor. […] My family motivates me. (FG16; age 41)

#### Children

Children, including grandchildren, were a large motivator for men’s health-seeking behaviors. In 14 of 40 (35%) focus groups, children were noted as influential in men’s decision-making. In contrast to the active motivation provided by women in the men’s lives telling them to “go” seek health care, the presence of children in their lives provided a more passive motivation. Children were often reported as motivators for men to seek care so that the men could live longer to see the younger generation grow up.

One thing about that, that’s one reason I will work smarter and go to the doctor, get preventive medicine, be checking on prostates and things of that nature. And I go to the doctor religiously. My wife always picks on me because she says, “You go to the doctor every year, same day.” Yes, I do. I want to make sure I’m around to see my kids get married, graduate high school, graduate college and continue on and have grandkids. So, my motivation is my family. (FG07; age 45)

What motivates me to go is my family. You know, my grandkids, because my life, I look at my life to be connected. I’m not just living for myself; I live for myself and my family. . . . That motivates me to go, to stay as healthy as I can, to try to live a clean life, do those things. If your family, your wife or your son or your daughter or your grandchildren seeing you sittin’ home sick and not doing anything about it, they wonder why. Why is it you’re not going to the doctor? It affects them. (FG04; age 62)

Men noted that their roles as examples for the children in their lives encouraged them to seek necessary medical care. They reported wanting to teach the children in their families to have positive health-seeking behavior by setting a good example.

What really motivated me to go to the hospital, like he said, your children. And to be open and honest with them and let them know what’s your status, what your situation is. This way they don’t run into the same things that we ran into in the streets out there. So it was important for me to go to the hospital to expose them that we can get medical care if needed and to prevent them from doing the things that we did, so I exposed that. (FG09; age 48)

For some men, the desire to teach and model more positive health-seeking behaviors for the children in their family was connected to their upbringing and the kinds of health influences they had growing up, from their parents or other relatives. Men referenced their upbringing either as the catalyst for positive health-seeking behaviors or as providing a perceived justification for negative health-seeking behaviors.

#### Parents

Parents had a substantially positive impact on men’s health-seeking behaviors as reflected in the discussions of 13 of the 40 (32%) focus groups. This parental impact was discussed as occurring through direct communication and active motivation. For example, men reported seeking medical care because parents instructed them to go, which reflected a positive influence.

That be my mom because when I call her and tell her [about my symptoms, she says], “Oh, you going to the doctor.” It’s the only reason I go because of her, other than that I wouldn’t even go nowhere. I’d stay in the house. (FG28; age 42)

Although parents’ influence was more often reported to be direct as in the manner already discussed, men reported seeking health care services based also on their (passive) observations of the illnesses or death of their parents. These observations and experiences provoked them to avoid similar health issues or circumstances by taking better care of their own health.

Yeah, well what motivates me is my father died at 51. And that had a major impact on my life and my entire family’s life. And so in my quest to live to be an old, old man, I know part of that quest is preventive care. (FG04; age 61)

#### Other relatives

Other relatives mentioned to have influence on participants’ health-seeking behaviors included siblings, grandparents, and extended family (i.e., aunts/uncles, cousins, nieces/nephews). These relatives or nonspecific references to family were noted in 21 of 40 (52%) focus groups. Some men reported being advised by relatives on their health or health care, but more frequently men highlighted that it was observing their extended family’s medical history that provided motivation for seeking medical care in an effort to avoid experiencing similar medical issues themselves.

As for me and my family, most of our conversations . . . it’s about goin’ to the doctor and taking care of yourself. We’re big on that. I have brothers that’s passed away. They wouldn’t take care of themselves. That’s why they’re gone, diabetics. I had one brother die from HIV. And now we’re like, “Hey, did you take that appointment?” And once you instill that and you groom and stay on it, you’ll probably have a better quality of life. (FG16; age 50)

#### Upbringing

In addition to the ways that parents and relatives positively influenced motivation for men’s health-seeking behaviors through instruction, advice, and family medical history, men also talked more generally about how their upbringing affected their beliefs and behaviors around seeking medical care in ways that inhibited health seeking. For example, for many, the concept or experience of growing up in families where seeking medical care was not greatly emphasized shaped their beliefs and behaviors toward medical care in adulthood.

I go back to that generalization, the generalization that within the black family foundation if there is not a show, like the mother and the father makin’ routine visits to the hospital, then that child’s not going to do it either. (FG29; age 62)

I guess being raised, my parents . . . I guess they didn’t enforce that, take me to the doctor and stuff. […] They didn’t go . . . my mom didn’t go a whole lot either, and she lives pretty . . . I mean she’s doing well, thus far been doing good. But I guess I just wasn’t raised like that. (FG01; age 47)

Men often noted that if they were not incapacitated by the symptoms of an illness or condition, they would ignore the symptoms and pain or cope with it through home remedies and self-medication. They indicated that even if they were in severe pain but could still continue their daily activities, particularly going to work, then they would treat the symptoms as best they could and keep on going. As one participant summarized, “If it ain’t broke, I’ll keep on pushin’. If I can get out of the bed, if I can take a shower and get out to work and work all day, I’m all right” (FG34; age 31). This mentality of “If it ain’t broke, I’m all right” was reported to be influenced by their upbringing in observing and hearing about the health-related practices and beliefs of relatives.

… as long you’re breathing and you woke up this mornin’, as long as God got you up this mornin’, then you’re fine. (FG10; age 43)

African American men’s experiences around medical care during upbringing, coupled with their families’ and communities’ use of home remedies as common treatment for ailments, can serve as a barrier for men seeking traditional medical care and preventive health services in their adulthood. Men spoke about their families’ use of home remedies and self-medication in lieu of going to the doctor for treatment of medical conditions, often in relation to socioeconomic status. Home remedies were viewed as a reliable alternative and less expensive than going to a doctor; for some it was conveyed as the best option, given families’ financial situations (e.g., large families, no insurance, high cost of medical care).

We had momma and big momma and anything wrong they had a cure. [. . .] So black men . . . and basically what we did is we could break our arm and momma would have it fixed. What hospital? Who’s got the money to take you to a hospital? Shoot, don’t go. It’s just like now, all of us sitting in here, if we twist our knee, our ankles playing ball we didn’t go to no doctor. We ain’t went to no doctor. Put an ace bandage on and get back on the court, still hurt. You could’ve had a broke ankle, and didn’t even know it, but our culture is just so self-preserving and so self . . . taking care of ourselves by doing it ourselves . . . I need a PhD . . . I got some remedies for you. (FG09; age 56)

It’s like I said, it’s a heritage thing. I mean, this is a learned process. And you might not think about it like that but it was taught to us and we taught our kids that things cost money, don’t bother. You got a toothache, take an aspirin, or get some ice and wrap your jaw up. You know what I mean? Home remedies, whatever it took. (FG11; age 51)

### Cultural and Gender Norms

In connection with upbringing, for those who did go to the doctor during childhood, mothers were most often noted as taking them to the doctor, whereas fathers were more commonly noted for the example they set for their sons during childhood. Men commented that their fathers were the “breadwinners” working to provide for the family and did not take time away from work to focus on health issues. Together, both parents had influence on health seeking as well as in how they socialized their sons, directly or indirectly. Some provided the message that going to the doctor was contradictory to the male gender norm of being tough and strong.

My father never talked to me about health. I never had my uncles or grandfather really wanna talk to me about my health. I guess due to them tryin’ to raise their kids and take to the responsibilities and stuff like that. […] I’m in a family of five boys and three girls, I remember when I used to do something wrong or run and fall down or hurt my knee, come to my father cryin’ he’d bust me upside the head, “Boy, stop cryin,’” [Chuckling] And my sister, she couldn’t come out and play with us. She was in the house or in the window. Sometimes when daddy and mom weren’t home they [sisters] were runnin’ with us to try to climb trees and they would get caught and my daddy would tell them, “You go in the house.” So from a child they were trained to watch us fall out and a man was trained to be macho, to overlook it, to say it don’t hurt and to keep on goin’. (FG27; age 52)

And a lot of times we look at it, dad never went to the doctor, and he worked hard out there. I’m working in a factory doing nothing, pushing a little forklift around, I ain’t going, I’m as tough as he is. So that goes back to that training and even mothers . . . said, “Toughen up, stop being a little girl and get out there.” (FG08; age 41)

Relatedly, these cultural gender norms contributed to a perception of invincibility that was frequently noted to factor into men’s decision-making about seeking health care. In 29 of 40 (72%) focus groups, men discussed invincibility—a perception of immunity to risk or hazard—as a factor affecting whether or how they seek health care. It was highlighted that younger men more often perceived themselves to be invincible.

You’re younger, you’re invincible. You think you’re invincible. I mean you can put up with the pain unless there’s some major broken bone or gunshot wound or stabbing or whatever. But just wake up in the morning with no pain, go to bed, no pain, go out through the day, no pain, no need to go to the doc. (FG01; age 52)

### Peer Influences

Peers contributed to men’s beliefs and behaviors around health care. In all 40 focus groups (100%), participants mentioned peer influence on their current health care seeking. Participants indicated that peers played a direct role in influencing health behaviors or beliefs by, for example, dispelling myths related to procedures or treatments. Peers contributed less directly by providing details of how others in their age cohort—childhood friends or classmates—had been diagnosed with or died of various conditions. Much of the influence from peers was of a positive nature, inspiring men to take their health more seriously, to view preventive measures more favorably, or to actually go to the doctor.

What kind of helps me . . . getting back to doing this, I’m talking to people in my age group [. . .] So that motivates me to go on and take the test. So by just talking to people in my generation, in my age group would make me to go. (FG23; age 52)

As far as when [I] go to the doctor now, like you were saying, the type of age that I am, and even some friends that’s around my age and they’ve got health problems. I’m like, “What in the world, you had a heart attack at 35? What is going on around here?” That kinda had me start thinking, “Well, maybe I do need to check myself.” (FG32; age 35)

Not all peer influences regarding health seeking were positive. Men also reported negative peer influences involving either hearing about a bad experience from a peer, hearing misinformation from a peer, or being influenced by stigmatizing comments made by peers. In particular, peers were implicated in reinforcing the previously mentioned male gender stereotypes of toughness and invincibility that precluded men’s admitting to or seeking care for health issues.

A lot of us do things or don’t do things because of the social environment where you at and the cultural thing and this peer pressure thing. “Man, you a little punk, you ain’t got to go to no hospital, it ain’t nothing but a cold.” It’s things like that. You know what I’m saying? “You acting like a little baby, you ain’t nothing but a little girl.” You know what I’m saying? You don’t want to hear that. So you avoid it. (FG06; age 45)

#### Community influences

Along with the influences from peers in a man’s community already detailed, focus group participants expressed receiving health-related social support from others in their community. Specifically, preachers, teachers, and celebrities—people typically held in high regard within the Black community—were reported to have influence on the health-seeking behaviors of African American men. Preachers and teachers were seen as taking personal interest in men, which helped influence behavior.

Just like last year I was in nursin’ school and we had to test each other’s blood pressure. Mine came back 165 over 135. My teacher told me to get the hell out of her classroom. She said it just like that, “Go to the doctor.” And I went, “No, I’ll be all right.” She was like, “No.” And if I don’t I wasn’t welcome back in the class. She told me to go to the hospital ’cause she said I was on the verge of having a stroke. And ever since then I’m on blood pressure meds. (FG38; age 32)

Public service announcements, mass media campaigns, and news stories featuring Black celebrities and health issues helped men in a different way, fostering the thought, “Well if he can do/does it, I can.”

The radio, Michael Baisden, he had influenced me to go check my prostate out, listening to him. On the media . . . I think I’ve seen one commercial with this African American male talking about prostate cancer and the effects and he was saying, “Had I checked it out earlier I would’ve known, I may have lived a little bit longer. (FG23; age 45)

## Discussion

Findings from this analysis confirm that social relationships are important influences on the health and health care–seeking behaviors of African American men and go further to delineate the nature of the influences across relationship types, both in direct provision of information and support and in the indirect messages they send. With family, female partners (i.e., spouses, girlfriends) provide encouragement and instruction for men (e.g., telling them to go) to seek medical care. The presence of a partner intensified men’s sense of responsibility to take care of themselves so as to be around for their partners. A similar motivation was derived from children; men reported taking care of their health in order to provide for and watch the development of their children (and grandchildren). Parents and other relatives positively influenced men’s beliefs and behavior through their own health problems, which inspired in participants the desire to maintain their health in order to avoid similar medical issues. Family—along with peers and culture—also provided negative influences on men’s health-seeking behavior, in the form of socialization around gender norms. Cultural gender norms often included emphasis on toughness in boys and men, which manifested for some into a perception of invincibility and/or an avoidance of medical care to avoid the appearance of weakness. Of note, African American men have the twofold burden of contending with male gender norms of machismo and stoicism and cultural norms of bias against using health care, both of which influence their health-seeking behaviors. Additionally, entangled with culture and gender norms was the crosscurrent of financial strain and limited ability to afford health care, the intersectionality (Griffith, 2012) of structural factors, which are not directly discussed here, but, which can be seen in several of the participants’ quotes within this article.

This study sample was not representatively drawn, which limits the generalizability of the findings; however, the high proportion of participants in the sample who were both unemployed and uninsured suggests that the study may have captured those men most at risk for suffering health disparities, as previous research shows that the daily hassles encountered by those in low-income brackets likely further delay health care seeking (Jacob, Arnold, Hunleth, Greiner, & James, 2014). Within this context, the present study findings suggest at least three potential avenues for approaching the issue of African American men’s health-seeking behavior.

### Build on Positive Personal Relationships

Given the positive and motivating influence of female partners and children noted in this study as well as other studies related to health communications and health promotion (Allen et al., 2018; Griffith, Ellis, & Allen, 2012), programs attempting to increase men’s health-seeking behavior can include these family members directly, either through joint activities or by enlisting partners and children in the dissemination of health behavior change communications (Friedman, Corwin, Rose, & Dominick, 2009; Owens, Jackson, Thomas, Friedman, & Hébert, 2015). In both of these cases, like those described for teachers and preachers, the personal connection can take ordinary health information and make it personal, if someone expresses concern and caring for a loved one’s health.

### Build on Positive Cultural and Gender Norms

Men repeatedly referenced the “breadwinner” or “caretaker” role they were socialized to fulfill, in relation to both partners and children. In conjunction with the suggestion already mentioned, programs could play on this gender norm to a public health advantage by developing health promotion messages that validate and celebrate the “responsibility” pieces of gender expectations—that you can’t take care of them if you’re not alive and healthy. This is the approach taken by Agency for Healthcare Research and Quality (AHRQ) in their “stubborn” campaign, as part of the “healthy men” project (AHRQ, 2012; Suennen, 2011). Similarly, program efforts could build on the cultural norms of self-sufficiency in health care (i.e., how home remedies are often used first) by emphasizing the similarities of self-sufficiency and the aim of prevention—to avoid needing treatment for medical issues. There is the potential to reframe preventive care as a way of maintaining self-sufficiency.

### Diffuse Negative Peer Influences With Positive Deviance

Changing gender norms is challenging, and particularly among peer groups. Rather than tackle it head-on, public health practitioners might again approach it by building on what is working to positively influence health. For example, male gender norms were a negative factor in peer relationships; however, in most cases, peers shared their knowledge about health issues, discussed myths and experiences around preventive screenings, or shared details of how others their age had been diagnosed with or died of various medical conditions. In a study of prostate cancer screening decision-making among urban African American men, Plowden (2006) reported that men stressed the importance of peer engagement in an “ideal” prostate cancer outreach program. Similarly, in a focus group study on male peer influence on middle-aged and older African American men’s motivation for physical activity, Griffith, King, and Allen (2013) reported that peer social support was a motivator for men to begin and maintain physical activity. Of note for the present study, several men in the focus groups commented about how helpful the focus groups themselves were for learning more and sharing health details with other men, providing a potential format for a peer-level intervention. Many men admitted that they came to the focus groups for the monetary reimbursement, but left happy to have learned something. Providing peer outlets for and validation of “positive deviance”—actions and behaviors that defy norms or stereotypes, yet lead to desired outcomes—would be another way to address this (Bonolo, Senger, Ramiro, & Araujo, 2014; Marsh, Schroeder, Dearden, Sternin, & Sternin, 2004). This could include peer-to-peer programs that provide a conducive environment for African American men to discuss health issues, share experiences, and hear accurate health information as another way to influence men’s “machismo” mentality when it comes to seeking medical care. An example of such peer-to-peer (brotherhood) health promotion is the Reclaim Our Strength national digital campaign by Henry Health in partnership with Alpha Phi Alpha Fraternity, Inc., a historically African American men’s intercollegiate Greek-letter fraternity, to raise awareness of the emotional and mental health of Black men as a public health priority (“Reclaim Our Strength,” 2018).

The findings from this study add detail to our understanding of the ways African American men recognize and act upon a sense of responsibility to their family, friends, and peers when engaging in health-seeking behaviors. Health promotion advocates and implementers can harness the positive influences of relationships and cultural norms to affect health-seeking behavior, and ultimately the health status of African American men experiencing health inequalities throughout the United States.
